# Nitrate Reductase-Mediated Nitric Oxide Regulates the Leaf Shape in *Arabidopsis* by Mediating the Homeostasis of Reactive Oxygen Species

**DOI:** 10.3390/ijms20092235

**Published:** 2019-05-07

**Authors:** Qiao-Na Pan, Chen-Chen Geng, Dan-Dan Li, Shi-Wen Xu, Dan-Dan Mao, Saima Umbreen, Gary John Loake, Bei-Mi Cui

**Affiliations:** 1 The Key Laboratory of Biotechnology for Medicinal Plant of Jiangsu Province, School of Life Science, Jiangsu Normal University, Xuzhou 221116, China; youyouwel@163.com (Q.-N.P.); gengcc1314@163.com (C.-C.G.); shiwenxu97@163.com (S.-W.X.); 15150080176@163.com (D.-D.M.); 2 Institute of Molecular Plant Sciences, School of Biological Sciences, Edinburgh University, Edinburgh EH9 3BF, UK; saima.umbreen@ed.ac.uk (S.U.); g.loake@ed.ac.uk (G.J.L.); 3 Haikou custom, Haikou 570311, China; 108074182@qq.com; 4 Transformational Centre for Biotechnology of Medicinal and Food Plants, Jiangsu Normal University—Edinburgh University, Xuzhou 221116, China; 5 Institute of Plant Protection, Jiangsu Academy of Agricultural Sciences, Nanjing 210014, China

**Keywords:** nitric oxide, reactive oxygen species, antioxidant enzymes, leaf development, nitrate reductase (NR)

## Abstract

As a gaseous biological signaling molecule, nitric oxide (NO) regulates many physiological processes in plants. Over the last decades, this low molecular weight compound has been identified as a key signaling molecule to regulate plant stress responses, and also plays an important role in plant development. However, elucidation of the molecular mechanisms for NO in leaf development has so far been limited due to a lack of mutant resources. Here, we employed the NO-deficient mutant *nia1nia2* to examine the role of NO in leaf development. We have found that *nia1nia2* mutant plants displayed very different leaf phenotypes as compared to wild type Col-0. Further studies have shown that reactive oxygen species (ROS) levels are higher in *nia1nia2* mutant plants. Interestingly, ROS-related enzymes ascorbate peroxidase (APX), catalases (CAT), and peroxidases (POD) have shown decreases in their activities. Our transcriptome data have revealed that the ROS synthesis gene *RBOHD* was enhanced in *nia1nia2* mutants and the photosynthesis-related pathway was impaired, which suggests that NO is required for chloroplast development and leaf development. Together, these results imply that NO plays a significant role in plant leaf development by regulating ROS homeostasis.

## 1. Introduction

Leaves are critical organs for the survival of plants as they are the primary source of photosynthesis-derived energy and also provide a large area for direct interaction with the environment [1,2]. Most importantly, the development of leaf shape and size contributes to the world food security. As settled organisms, plants have evolved a sophisticated molecular mechanism for the development of leaf shape and size [3,4]. Despite a huge diversity in shape and size, early leaf developmental modules are almost conserved in angiosperms [5,6]. In this context, leaf shape and size are initially determined by the meristematic cell proliferation and then by cell expansion. Co-ordination between cell proliferation and cell expansion is critical for the development of the final size of the leaves. Mutations in the genes that control polar cell proliferation and cell expansion have yielded short leaves [7,8]. In contrast, transgenic lines expressing *ICK1* (cyclin-dependent kinase inhibitor 1) or *KRP2* (Kip-related protein 2), both of which inhibit the proliferation of leaf cells by interacting with CDKA–cyclin complexes, resulted in reduced cell number and smaller leaf size [9]. Therefore, plant leaf shape is dependent on temporal and spatial distributions of cell proliferation and expansion, both of which are regulated by multiple molecular pathways [3]. For example, phytohormones such as DEL1 regulate plant growth and development by modulating cell proliferation [10]. In addition to phytohormones, redox signaling molecules, such as ROS (reactive oxygen species) and NO [11], have been shown to play crucial roles in various physiological processes including plant leaf development [12,13,14]. Although leaf development has been the subject of numerous studies, the molecular mechanism that controls it remains far from understood.

The cellular redox status plays an important role in the regulation of cell fate and organ development, and changes in redox status are known to occur during cell proliferation and expansion [15,16]. Increasing reports suggest that leaf shape development is regulated through the modification of redox status within plant cells [17]. In this context, *Arabidopsis* mutants with higher ROS production (such as *kua1*) and higher NO generation (such as *nox1* and *gsnor1-3*) have smaller leaves as compared to wild type plants [8,18], which strongly suggests that redox signals play a central role in plant leaf development. Many biochemistry studies have suggested that redox molecules, including RNS (reactive nitrogen species) and ROS, modulate the activities of proteins by oxidation of amino acids such as Cysteine (Cys), Tryptophan (Trp), and Serine (Ser), which is a prominent feature of redox signaling networks [16]. For example, TCP1 (TEOSINTE BRANCHED1-CYCLOIDEA-PROLIFERATING CELL FACTOR1) transcription factors are well known to regulate plant leaf development associated with cell proliferation and growth [19]. Specifically, a conserved Cys20 within TCP1s could be a target of H_2_O_2_- and NO-derived oxidation, which inhibits its DNA binding activity during plant development [19]. Numerous redox-sensitive proteins have been identified, which shows the role of redox regulation in plant development [14]. Since the redox status plays a central role in plant development, imbalance of redox homeostasis will always result in unfavorable conditions. To cope with this, plant cells have developed enzymatic and non-enzymatic systems to maintain the appropriate redox status. Furthermore, high levels of NO could provide a feedback to inhibit NADPH oxidase activity through S-nitrosylation of AtRBOHD (*Arabidopsis thaliana* Respiratory Burst Oxidase Homologue D) at Cys890, and thus reduce production of reactive oxygen intermediates (ROIs) during immune response [20]. These evidences suggest that the oxidative status generated by reactive oxygen species seems to be alleviated by reducing ROS production. Therefore, we need more powerful genetic tools to further explore their roles in plant development.

So far, redox molecules such as H_2_S, ROS, CO, and NO have been shown to have functions in various physiological processes among plants and animals [13,21,22,23,24]. Moreover, H_2_S was shown to regulate ROS and NO levels in BV2 microglial cells [22]. In plants, RNS and ROS synthesis is a routine requirement for plant development. A major source of NO production in plants is nitrate reductase (NR), which also facilitates its homeostasis [25]. In *Arabidopsis*, NR is encoded by two genes *NIA1* and *NIA2*. NR double mutant *nia1nia2* failed to synthesize NO [26,27]. Interestingly, NR-dependent NO plays a crucial role in plant development and various stress responses. For example, NR-mediated NO is essential for abscisic acid(ABA)-induced stomatal closure, floral transition, and root hair development, and NR-dependent NO also plays a role in auxin-induced NO production [28,29,30,31]. Besides this, NR-dependent NO regulates various abiotic stresses such as freezing, hypoxic, and osmotic stress tolerance [32,33,34,35,36], as well as biotic stress responses to *Pseudomonas syringae* [37].

The accumulating evidence suggests that NO and ROS can function independently or synergistically to regulate development and stress responses [12,38,39,40]. However, until now, the cross-talk of NO and ROS signals in plant leaf development remains to be uncovered. In this study, we employed the NR-deficient mutant *nia1nia2* to investigate how NO mediates leaf development, and how the cross-talk between ROS and NO regulates it. We found that NO is required for leaf development. Further, ROS levels in *nia1nia2* mutants were increased, but the activities of ROS-related enzymes APX (ascorbate peroxidase), CAT (catalases), and POD (peroxidases) were reduced as compared to wild type Col-0 (Columbia-0). Our findings emphasize the role of NO in leaf development and also the importance of ROS homeostasis to regulate it.

## 2. Results

### 2.1. Lack of NR-Mediated NO Production Affects the Leaf Shape and Size in Arabidopsis

**Figure 1:**
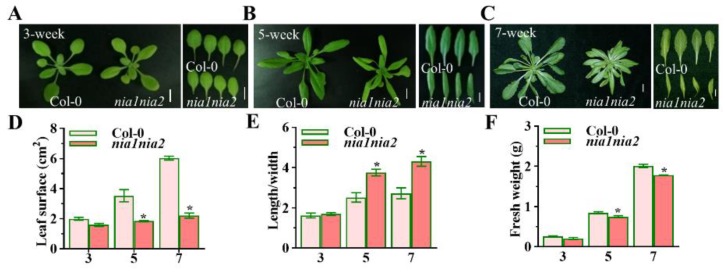
*nia1nia2* mutants showed altered leaf shape development. (**A**–**C**), Leaf phenotypes of Col-0 and *nia1nia2* plants at 3, 5, and 7 weeks. Scale bar, 2 cm. (**D**), Measurement of average leaf surfaces of Col-0 and *nia1nia2* plant. Leaves were detached from 3, 5, and 7-week-old plants and then measured. Eight plants of each ecotype were used for measurement, and error bars represent standard deviation (SD). (**E**), Leaf length-to-width ratios of 3, 5, and 7-week-old Col-0 and *nia1nia2* plants were measured. Error bars represent SD from eight replicates. (**F**), Fresh weight of 3, 5, and 7-week-old plants of Col-0 and *nia1nia2*. Error bars represent SD from eight replicates. *, *p* < 0.001 (Student’s *t*-test). Experiment was repeated three times with similar results.

In order to study the role of NR-mediated NO in *Arabidopsis* leaf development, we selected *nia1nia2* mutant lines for conducting the research. We characterized the leaf shape and size at three various time points (3-, 5-, and 7-week-old plants) of the plant life cycles in order to understand the differences in leaf shape and size at various stages of leaf development. Our results showed that there is not much difference in leaf shape or size of 3-week-old *nia1nia2* plants as compared to wild type Col-0 (Figure 1A). However, 5-week-old plants of *nia1nia2* mutants have much narrower and smaller leaves as compared to wild type Col-0 (Figure 1B). Interestingly, leaf shape and size differences became even more obvious at 7 weeks with the development of rosette leaf shapes in *nia1nia2* mutant plants, which were strikingly different to wild type Col-0 (Figure 1C).

Next, we quantified the leaf shape and size by measuring leaf surface area, and calculated the leaf length-to-leaf width ratios at the same three time points. Our data have suggested that leaf surface area is significantly reduced in *nia1nia2* mutants as compared to wild type Col-0 plants at all three time points (Figure 1D). However, leaf length-to-width ratios are significantly different between *nia1nia2* mutants and wild type Col-0 plants at 5 and 7 weeks only (Figure 1E). Further, *nia1nia2* showed significantly less fresh weight than Col-0 in 7-week-old plants (Figure 1F).

In summary, our data have clearly indicated that NR-mediated NO generation might be required for leaf development, especially during later stages of leaf development.

### 2.2. nia1nia2 Mutant Plants Have Smaller Leaves as Compared to Wild Type Col-0 due to Having Lesser Number of Cells and Reduced Cell Size in Their Leaves

**Figure 2:**
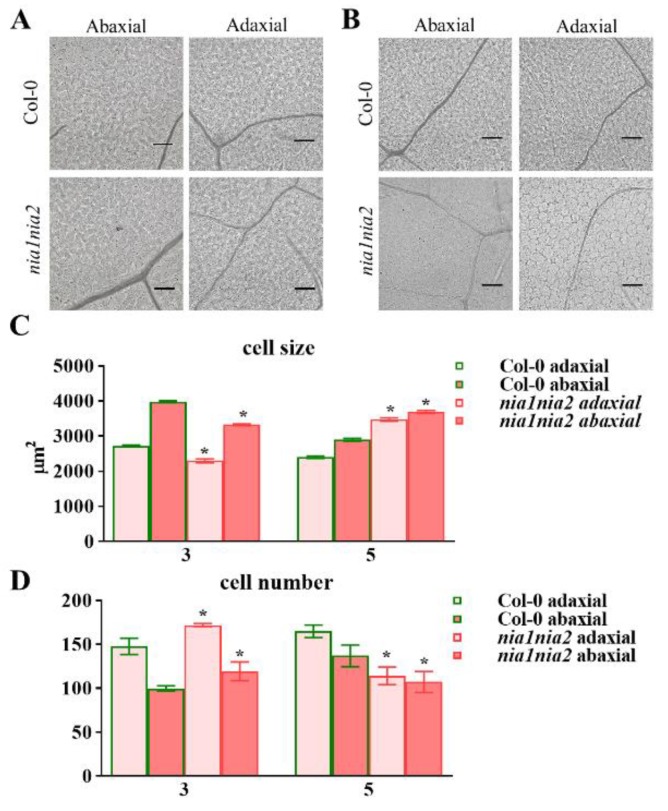
The *nia1nia2* mutation affects cell size and cell number. (**A**,**B**) Adaxial and abaxial epidermal cells in 3-week- (**A**) and 5-week-old plants (**B**) of Col-0 and *nia1nia2*. Scale bar, 100 μm. (**C**,**D**) The size (**C**) and number (**D**) of epidermal cells in Col-0 and *nia1nia2*. Error bars represent SD from six replicates. *, *p* < 0.05 (Student’s *t*-test).

Leaf size and shape is determined by the sum of cell number and cell size. In order to investigate whether differences in size and shape of NO-deficient mutant *nia1nia2* leaves are due to cell proliferation, expansion, or both, we examined the cell number and cell size within adaxial and abaxial epidermal cells of the third and fourth rosette leaves of 3- and 5-week-old plants. Our results showed that the cell size of adaxial epidermis in *nia1nia2* plants was slightly smaller. However, the cell size of abaxial epidermis is significantly smaller as compared to Col-0 in 3-week-old plants (Figure 2A,C). Both abaxial and adaxial epidermis have more cells in *nia1nia2* plants than Col-0 in 3-week-old plants (Figure 2A,D). Interestingly, in 5-week-old plants, the cell size of *nia1nia2* was significantly larger than that of Col-0 in both upper and lower epidermal cells. However, the count of cell number in both adaxial and abaxial epidermis was less in *nia1nia2* mutant plants as compared to Col-0 (Figure 2B,C).

Collectively, NO has affected both cell size and cell number during leaf development in *nia1nia2* mutant plants as compared to Col-0, demonstrating that NO regulates leaf development in *Arabidopsis* by mediating cell number and size.

### 2.3. nia1nia2 Plants Have Lesser Chlorophyll a/b Contents as Compared to Col-0

**Figure 3:**
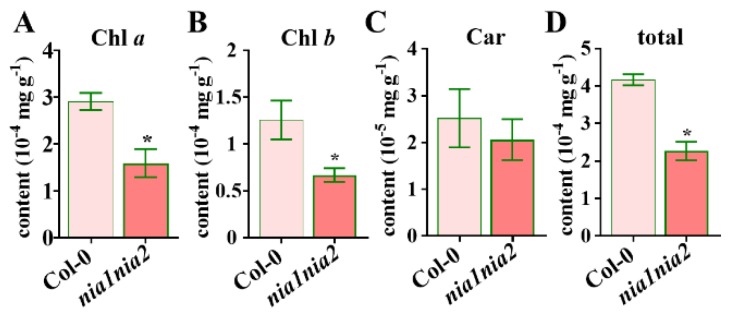
Analysis of chlorophyll and carotenoid contents in 5-week-old Col-0 and *nia1nia2* lines under normal growth conditions. The chlorophyll a (**A**), chlorophyll b (**B**), carotenoid (**C**), and total chlorophyll (**D**) were assayed in the detached leaves. Error bars represent SD from eight replicates. *, *p* < 0.05 (Student’s *t*-test).

NO is mostly produced inside the chloroplasts of the plants, which indicates that NO could regulate chloroplast development [14]. Interestingly, the leaf color of 5-week-old *nia1nia2* mutant plants is strikingly different than wild type Col-0. This observation has led us to analyze the chlorophyll (Chl) and carotenoid (CAR) composition in these leaves. Consistent with the observed color phenotype, the levels of Chl *a* and Chl *b* decreased significantly in *nia1nia2* mutants as compared to Col-0 in 5-week-old plants (Figure 3A,B). Consistently, the total Chl synthesis in *nia1nia2* leaves was inhibited (Figure 3D). Strikingly, carotenoid contents were similar between *nia1nia2* and Col-0 (Figure 3C), indicating that NR deficiency-derived NO production does not affect carotenoid synthesis. Collectively, contents of Chl *a*/*b* were changed in *nia1nia2* plants as compared to Col-0 plants, which has yielded differences in the leaf color.

### 2.4. NR-Generated NO Regulates Leaf Shape by Mediating ROS Levels

**Figure 4:**
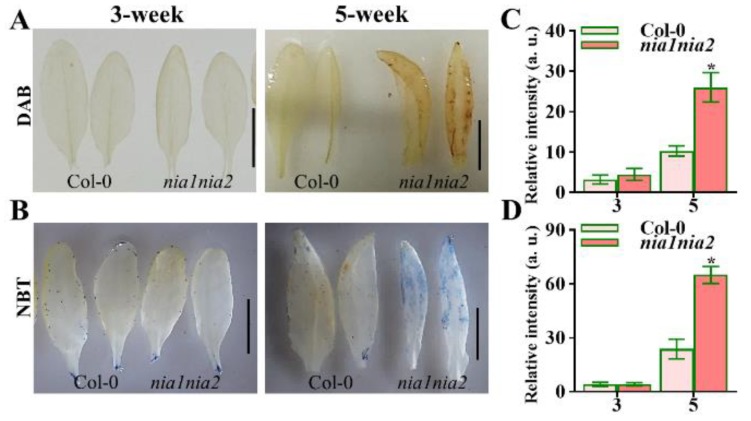
Analysis of reactive oxygen species (ROS) levels in Col-0 and *nia1nia2*. (**A**,**B**) Staining of indicated genotype leaves with 3,3-diaminobenzidine (DAB) (**A**) or nitro blue tetrazolium (NBT) (**B**) to reveal the levels of H_2_O_2_ and O_2_^−^, respectively. Scale bar, 1 cm. (**C**,**D**) Quantification of relative intensities of H_2_O_2_ (**C**) and O_2_^−^ (**D**). *, *p* < 0.05 (Student’s *t*-test).

ROS have been reported to regulate cell expansion and cell number, which determine final leaf shape and size [41,42]. NO has been shown to prevent oxidative stress through regulating ROS levels [24]. In order to test if NO is regulating leaf shape through mediating ROS levels in *nia1nia2* plants, we quantified ROS (hydrogen peroxide, H_2_O_2_ and superoxide, O_2_^−^) levels in 3-week- and 5-week-old plants. We stained 3-week-old plants’ leaves with 3,3-diaminobenzidine (DAB), which is a standard dye used to quantify the levels of H_2_O_2._ We found an increased intensity of DAB in 3-week-old plants of *nia1nia2* mutants leaves as compared to Col-0 (Figure 4A), suggesting that *nia1nia2* leaves contained slightly higher H_2_O_2_ levels than Col-0 at this stage of leaf development. Similar results were obtained when we quantified the intensity of DAB staining in 5-week-old plants (Figure 4A). Interestingly, the DAB staining showed that the H_2_O_2_ levels were markedly higher in *nia1nia2* mutants as compared to Col-0 in 5-week-old plants. Quantification of H_2_O_2_, as shown in Figure 4C, also supported the observation that *nia1nia2* mutant plants have higher production of H_2_O_2_ as compared to Col-0 in both 3-week- and 5-week-old plants.

In addition to the quantification of H_2_O_2_, we also quantified the superoxide (O_2_^−^) by nitro blue tetrazolium (NBT) staining, and as shown in Figure 4B, the O_2_^−^ levels were similar between *nia1nia2* mutants and Col-0. Moreover, the O_2_^−^ levels were higher than that in Col-0 in 5-week-old plants (Figure 4B). The quantification of NBT staining also suggested that there is a significant increase of O_2_^−^ in 5-week-old *nia1nia2* plants as compared to Col-0 (Figure 4D).

Taken together, these data suggest that the ROS levels (H_2_O_2_ and O_2_^−^) have increased in *nia1nia2* mutants as compared to Col-0, implying that NR-dependent NO production is required for ROS homeostasis during leaf development.

### 2.5. nia1nia2 Has Reduced Antioxidant Enzyme Activity

**Figure 5:**
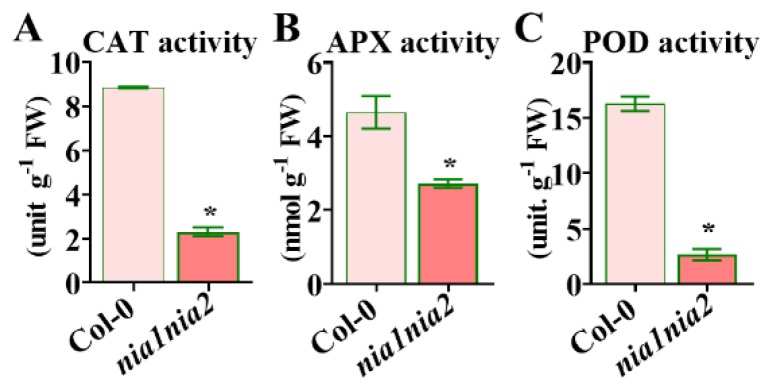
Activities of ROS-related enzymes in mutant *nia1nia2* and its wild-type Col-0. Third rosette leaves from 5-week-old plants of the indicated genotypes were collected for catalase (CAT)_ (**A**), ascorbate peroxidase (APX) (**B**), and peroxidase (POD) (**C**) activity assays. Vertical bars represent SD (*n* = 5). *, *p* < 0.05 (Student’s *t*-test).

High levels of ROS are harmful to cells, and so plants have developed a protective system to reduce the oxidative stress and avoid damage. The enzymes POD, CAT, and APX are ROS scavengers in the anti-oxidant protection system. As shown above, the ROS levels increased in *nia1nia2* mutants as compared to Col-0, and we further assayed the activities of these three ROS scavengers. As shown in Figure 5, the activity of CAT in leaves of 5-week-old *nia1nia2* plants was extremely reduced as compared to that of Col-0 plants. Furthermore, the activities of APX and POD in *nia1nia2* plants were significantly lower than that of Col-0 plants. Thus, it appears that inhibition of these antioxidant enzymes’ activities in *nia1nia2* mutant plants leads to an increase in ROS levels.

Collectively, this observation clearly suggests that APX-, CAT-, and POD-mediated ROS metabolism is relatively impaired in *nia1nia2* mutants, which has eventually altered their leaf morphology.

### 2.6. RNA Sequencing Showed Clear Differences in Key Gene Expression Levels in nia1nia2 Mutant Plants

**Figure 6:**
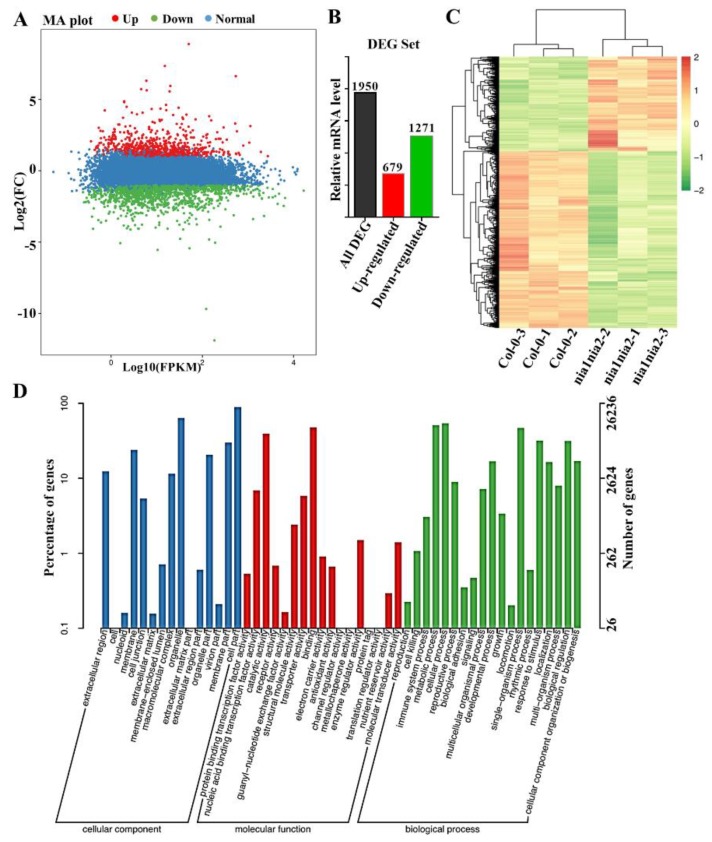
Nitrate reductase (NR)-deficient mutant *nia1nia2* plants have affected gene expression. (**A**) MA plot of RNA-seq data showing the up-regulated (red) and down-regulated (green) genes in *nia1nia2* plants compared with Col-0. (**B**) A total of 1950 TFs were identified at a Q-value < 0.05 in the transcriptome. Among these, 679 were up-regulated and 1271 were down-regulated (≥log2-fold change). (**C**) Heat-map showing the expression patterns of transcriptome between *nia1nia2* plants and WT. (**D**) Distribution of differentially expressed genes using pairwise comparisons in WT and *nia1nia2* plants.

Once we have established the role of NR-mediated NO production in leaf development of *Arabidopsis* through the regulation of ROS levels, we further conducted RNA sequencing to investigate the differences in gene expressions between *nia1nia2* mutant plants and Col-0 which could possibly explain the differences in leaf shape between these two genotypes. For this purpose, we compared the transcriptomic profiles of third rosette leaves of 5-week-old *nia1nia2* mutant plants and wild type Col-0 plants using RNA sequencing (RNA-Seq). We sequenced the libraries on IIIumina HisSeq 2000, and a total of 271,323,530 high-quality reads were generated. We found a difference in expression levels of a total of 1950 genes between these two genotypes. Interestingly, among these genes, 679 genes were up-regulated and 1271 got down-regulated (Figure 6A,B), and these genes were divided into two groups using a hierarchical clustering algorithm (Figure 6C). The global transcriptome changes in examined samples were further categorized based on their gene ontology (GO) and divided into groups of predicted or experimentally defined biological processes, molecular functions, and cellular components (Figure 6D). Functional categorization of differentially regulated genes revealed that a wide variety of biological processes are associated with pigment binding, and chloroplast and cell wall development.

**Figure 7:**
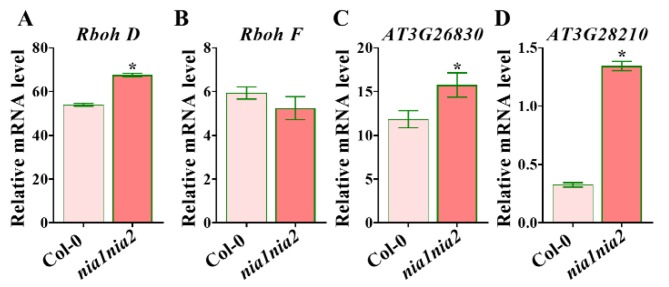
ROS relative gene expression levels in Col-0 and *nia1nia2* plants. RNA was extracted from indicated genotypes, and real-time PCR was performed. *UBQ10* was used as an internal control. Error bars represent SD from three biological replicates. *, *p* < 0.05 (Student’s *t*-test).

Interestingly, ROS synthesis key gene *RBOHD*, but not *RBOHF*, was highly induced in *nia1nia2* mutants as compared to Col-0, (Figure 7), in agreement with the fact that ROS production is higher within *nia1nia2* leaves as compared to Col-0. Additionally, mRNA levels of ROS response marker genes were increased in *nia1nia2* compared with Col-0 (Figure 7), suggesting that these ROS-related genes are induced in *nia1nia2* plants. Overall, transcriptome analysis gives more insight to understand the molecular mechanism underlying leaf shape in *nia1nia2* plants.

Leaf photosynthesis ability is mainly associated with Chl contents and leaf development [43]. Our RNA-seq data have shown a reduction in the expression of photosynthetic genes such as *Lhca* and *Lhcb*, and also *Psb* and *Psa* genes. This clearly indicates that the chloroplast and photosynthesis systems are severely compromised in *nia1nia2* mutant plants. Collectively, these data clearly suggest that NO might have a role in the reprogramming of chloroplast-related gene expression to alter Chl contents and, by extension, leaf development.

## 3. Discussion

NO is a key redox-active molecule of cellular signal transduction networks, which regulates many physiological processes in plants, including development and immunity [14,18,44,45], and also in animals, including immunomodulatory responses and oncogenesis [21,46]. However, little is known about the role of NO in leaf development. In mammals, NO is generated by nitric oxide synthase (NOS), a NADPH-dependent enzyme [47,48]. NOS catalyzes the conversion of L-arginine to citrulline and NO [48]. Despite several completed genome projects, the gene for NOS has not been uncovered in plants yet [11,49]. NR has been the main source of NO production in plants to date [30,31,32]. In *Arabidopsis*, NR occurs in the cytosol and is encoded by two genes: *NIA1* and *NIA2* [33]. In this study, we found that the NO-deficient mutant *nia1nia2* displayed distinguished leaf phenotype compared with Col-0, as shown in previous reports where *noa1*/*nos1* NO-deficiency mutations in *Arabidopsis* or rice affect the leaf development [50,51,52]. However, the underlying mechanism was left to be explored.

We observed that *nia1nia2* leaves were smaller in size as compared to Col-0 (Figure 1 and Figure 2). A relationship between leaf size and photosynthesis has been suggested in the literature [33]. Chloroplasts are the major source for free energy transduction (photophosphorylation) in plants, and NO has been shown to affect the function of chloroplasts through the inhibition of photophosphorylation [53]. NO affects photosynthesis and photorespiration in various plants [53,54]. For example, *OsNOA1* loss-of-function mutations in rice reduced chlorophyll levels during low temperature [55]. It was also reported that the application of a NO donor, SNP, could enhance the photosynthetic activity and reduce the Chl degradation [56]. By contrast, NO scavenger cPTIO reduced the SNP-enhanced Chl stability in bananas [57]. Further, NO is produced in chloroplasts [58], most probably through arginine and in a nitrite-dependent NO manner [59]. Our results have shown that *nia1nia2* mutant plants have lesser chlorophyll contents as compared to Col-0 (Figure 3). Consistently, another NO-related mutant, *noa1*, which had impaired NO production in stress responses, also showed similar phenotypes [52]. Further, NO-overproduction mutant *nox1* (CUE1) and *gsnor1-3/par2-1* showed stunted phenotypes and Chl synthesis [18,60]. Our RNA sequence data have shown that photosynthetic genes are down-regulated in *nia1nia2* mutant plants, which demonstrates that NO is required for Chl synthesis and, by extension, for proper leaf size development. These findings strongly demonstrated that the endogenous NO level is important for plant leaf development.

ROS regulate the leaf development by controlling cell proliferation and cell size [16]. In this study, we found that *nia1nia2* mutant plants have smaller leaves due to reductions in cell number and cell size as compared to wild type plants. Interestingly, our data have suggested that NO is regulating leaf size through controlling ROS homeostasis, as ROS synthesis gene *RBOHD* was highly induced in *nia1nia2* plants as compared to Col-0, as revealed by RNA-seq. We further confirmed this by qPCR studies (Figure 7). Hence, NO regulates ROS homeostasis through dual mechanisms: First, by enhancing ROS production (Figure 4 and Figure 7), and secondly by inhibition of ROS-scavenger enzymes, such as APX, CAT, and POD (Figure 5).

Past reports have shown that NR-dependent NO production is required for abiotic stress-induced antioxidant defense in wheat and bean plants [61,62]. It was shown that NO scavengers reduced the activities of all antioxidant enzymes under abiotic stresses, while SNP reduced the production of ROS, consistent with our results. Hence, our results have confirmed that NR-dependent NO mediates the leaf development and stress response by regulating ROS hemostasis.

In conclusion, our results presented here have demonstrated that NO-deficient mutant *nia1nia2* plants showed visibly different leaf phenotypes compared with WT Col-0 due to compromised cell proliferation. We have uncovered the mechanism behind this difference in leaf phenotype between *nia1nia2* mutant plants and wild-type plants: NR-dependent NO regulates leaf development by controlling ROS homeostasis in these plants. Indeed, this study sheds light on the critical role of NO in regulating plant leaf development.

## 4. Materials and Methods

### 4.1. Plant Growth and Morphological Analysis

*Arabidopsis thaliana* Columbia-0 (Col-0) and *nia1nia2* mutant plants used in this study [26] were grown on soil after cold stratification for 2–3 days under 10 h photoperiod conditions at 22 °C. To measure the leaf area, and width and length of indicated leaves, leaves were cut and photographed, and then stated measurements were performed with image J software.

### 4.2. Cell Number and Size Analysis

Leaves of indicated genotypes were bleached with clearing solution (8 g chloral hydrate, 3 mL water, and 1 mL glycerol), and then observed under microscopy [63]. Photos were taken at the same area of each leaf for both the abaxial and adaxial sides, and then the cell size and number were scored. At least 10 leaves of each genotype at given developmental stages were observed.

### 4.3. Staining and Analysis of H_2_O_2_ and O_2_^−^

For DAB and NBT staining, dyes were prepared following a previously described method with some modifications [64]. Briefly, the third rosette leaf of each genotype at a particular developmental stage was cut, and then stained in the DAB solution (1 mg/mL) for 8 h in dark for detection of H_2_O_2_, or NBT solution (0.5 mg/mL in PBS) for 3 h in dark for detection of O_2_^−^, followed by destaining of the leaves with ethanol until there was proper visualization of the stain. The final staining intensity was recorded by photographing the leaves. The H_2_O_2_ and O_2_^−^ contents were assayed as described previously [65].

### 4.4. Antioxidant Enzyme Extraction and Quantification

Enzymes were extracted from 0.2 g of leaf tissues of each genotype at a particular developmental stage using a mortar and pestle with 2 mL of extraction buffer containing 50 mM K-phosphate buffer (pH 7.6) and 0.1 mM Na_2_-EDTA. The homogenate was centrifuged at 15,000 *g* for 15 min at 4 °C, and the supernatant fraction was used to assay various enzymes according to the method described elsewhere [66].

### 4.5. Quantification of Chl Contents

For chlorophyll determination, the first two true leaves of each genotype were collected, and their fresh weights were determined individually. Leaves were extracted with 80% ethanol at 4 °C overnight with avigation under dark. Chl contents were determined as described in reference [67].

### 4.6. RNA Sequencing and Gene Ontology (GO) Enrichment Analysis

For transcriptome analysis, 5-week-old leaves from Col-0 and *nia1nia2* plants were grown in growth room. Total plant RNAs were extracted using the plant RNA isolation kit (Agilent, Santa Clara, CA, USA). A total of 3 ug of high-quality RNA per sample was used for sequencing on an Illumina HisSeq2500 platform, and 125 bp paired-end reads were generated. Reference genome and gene model annotation files were downloaded from a genome website directly (ftp://ftp. arabidopsis.org/home/tair) using TopHat v2.0.12. Cuffquant and cuffnorm (v2.2.1) were used to calculate the FPKM (Fragments Per Kilobase of transcript per Million fragments mapped) of genes in each sample [68].

Gene ontology (GO) enrichment analysis of differentially expressed genes was implemented by the GOseq R package, in which gene length bias was corrected. GO terms with corrected *p* value < 0.05 were considered significantly enriched by differentially expressed genes.
